# Composition and Diversity of Soil Fungi in Dipterocarpaceae-Dominated Seasonal Tropical Forests in Thailand

**DOI:** 10.1264/jsme2.ME17168

**Published:** 2018-05-30

**Authors:** Sarasa Amma, Hirokazu Toju, Chongrak Wachrinrat, Hirotoshi Sato, Akifumi S. Tanabe, Taksin Artchawakom, Mamoru Kanzaki

**Affiliations:** 1 Division of Forest and Biomaterials Science, Graduate School of Agriculture, Kyoto University Kitashirakawa Oiwake-cho, Sakyo-ku, Kyoto, 606–8502 Japan; 2 Center for Ecological Research, Kyoto University 509–3, 2-chome, Hirano, Otsu, Shiga, 520–2113 Japan; 3 Precursory Research for Embryonic Science and Technology (PRESTO), Japan Science and Technology Agency Kawaguchi, Saitama 332–0012 Japan; 4 Department of Silviculture, Faculty of Forestry, Kasetsart University Bangkok Thailand; 5 Department of Environmental Solution Technology, Faculty of Science & Technology, Ryukoku University Seta-Oe, Otsu, 520–2194 Shiga Japan; 6 Department of Biology, Graduate School of Science, Kobe University 1–1 Rokkodai, Nada-ku, Kobe, 657–8501 Japan; 7 Sakaerat Environmental Research Station, Wang Nam Khiao Wang Nam Khiao-District, Nakon Ratchashima, 30370 Thailand

**Keywords:** community structure, seasonal tropical forest, microbial community, soil fungi, high-throughput sequencing

## Abstract

Although fungi play essential roles in nutrient cycles and plant growth in forest ecosystems, limited information is currently available on the community compositions of soil fungi in tropical forests. Few studies have examined fungal community structures in seasonal tropical forests, in which forest fires potentially have a large impact on above- and belowground community processes. Based on high-throughput sequencing technologies, we herein examined the diversity and community structures of soil fungi in dry seasonal tropical forests in Sakaerat, northeast Thailand. We found that fungal community compositions diverged among dry evergreen, dry deciduous, and fire-protected dry deciduous forests within the region. Although tree species diversity did not positively correlate with soil fungal diversity, the coverage of an understory bamboo species (*Vietnamosasa pusilla*) showed a strong relationship with fungal community structures. Our community ecological analysis also yielded a list of fungi showing habitat preferences for either of the neighboring evergreen and deciduous forests in Sakaerat. The present results provide a basis for managing soil fungal communities and aboveground plant communities in seasonal tropical forests in Southeast Asia.

Fungi play essential roles in terrestrial ecosystems, organizing biodiversity and various ecosystem processes (37). Wood-decaying fungi decompose organic materials, thereby recycling nutrients within ecosystems (26). Plant pathogenic fungi attack specific host plants, which contributes to the maintenance of plant species diversity through negative “plant-soil feedback” (4). Mycorrhizal fungi assist plants in acquiring soil nutrients, such as phosphorus and nitrogen, in exchange for the photosynthetic products allocated from their host plants (48). Moreover, mycorrhizal fungi form common mycelial networks, which interconnect conspecific and heterospecific plant individuals belowground, increasing/decreasing the growth of seedlings (35, 46). Ectomycorrhizal fungi are associated with plant species in Pinaceae, Fagaceae, and Dipterocarpaceae, which often dominate forests in temperate and tropical regions (5). An understanding of these plant-fungus interactions is of particular importance in Southeast Asia, in which Dipterocarpaceae plants are major targets of forestry (54).

In forests in Southeast Asia, fungi play essential roles in belowground nutrient uptake by plants. Mycorrhizal fungi and other soil fungi have access to a significant proportion of nitrogen and phosphorus in tropical forests, in which heavy rainfall and highly weathered ultisols consisting of nutrient-fixing clays prevent nutrient uptake by plants (10). Therefore, in these forest ecosystems, close associations with belowground fungi are crucial for the survival and growth of vascular plants (25, 39). Based on these tight plant-fungus interactions, an understanding of the diversity and composition of soil fungal communities is of particular importance for managing forest ecosystems in Southeast Asia.

While recent studies revealed the community structure of belowground fungi in tropical rain forests in Southeast Asia (10, 43), limited information is currently available on soil fungi in seasonal tropical forests, which are strongly influenced by annual forest fires in the dry season (16). Frequent fires modify the forest structure (52) by changing the biomass of young vulnerable trees: for example, many tropical dry forests have been converted to grasslands by fire (24). Moreover, forest fires not only reduce the supply of carbon and nitrogen in soil (19, 64), they also affect the soil fungal community structure (7), changing nutrient cycles in forest ecosystems. Despite these potential impacts of forest fires on not only aboveground, but also belowground ecosystem processes (65), only limited information is currently available on the community compositions of soil fungi in seasonal tropical forests.

The present study provides the first inventory data of soil fungal communities in two major types of tropical seasonal forests in Thailand. In a dry evergreen forest (DEF) and dry deciduous forest (alternatively dry dipterocarp forest or deciduous dipterocarp forest) (DDF) in Northeast Thailand, we sampled soil specimens and revealed fungal community structures based on pyrosequencing. We then compared fungal community structures among the forest types and identified fungi unique to either of the forest types and those common to both forest types. We also investigated whether fungal community structures correlated with aboveground plant community structures. The present results provide a basis for clarifying how belowground soil fungi contribute to whole ecosystem processes in seasonally dry tropical forests in Southeast Asia.

## Materials and Methods

### Study area

**Fig. 1:**
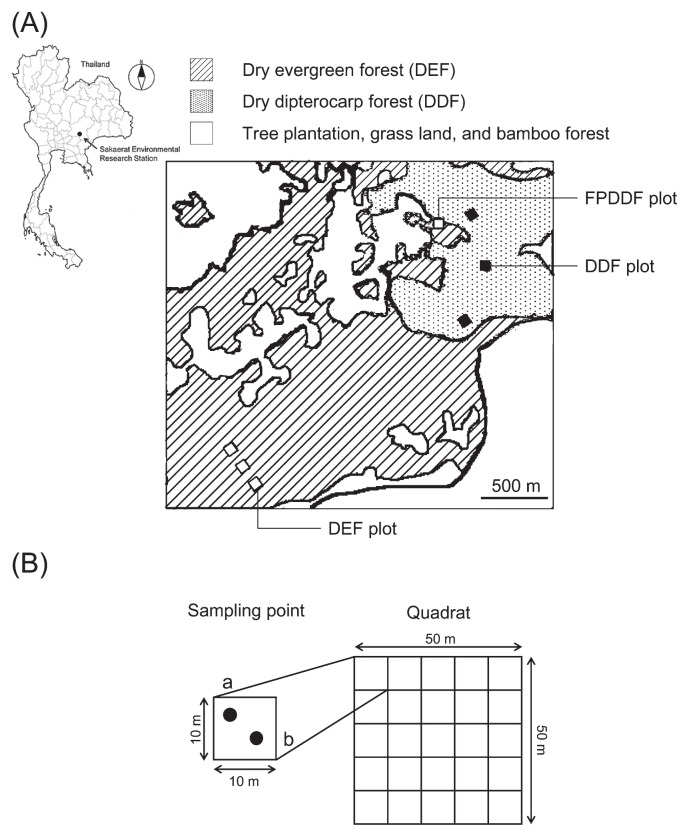
Information on research sites. (A) Location map of research sites in 2003 (Takuya Saruwatari, unpublished); (B) Illustration of soil sampling. Long-term fire protection at the forest patch, including the FPDDF plot, was identified as DEF, although canopy species mostly consisted of deciduous species in this map.

The present study was conducted in the Sakaerat Environmental Research Station (SERS) in the Nakorn Ratchasima Province, Northeast Thailand (14°30′N, 101°56′E) (Fig. 1) (34). The climate of this area is classified as tropical savanna (Aw) according to Koppen’s criterion. The annual mean temperature was 26.4°C and mean annual precipitation was 1,009 mm for 2002–2012 (http://www.tistr.or.th/sakaerat/Meteorlogical.htm). There is a distinct dry season between November and February, in which monthly rainfall is less than 50 mm. The altitude of the area ranges between 250 and 762 m and its topography is mountainous with 10–30% slopes. The soil is generally poorer in nutrients and characterized by more rock outcrops in DDF than in DEF. The soil of this area is underlain by sandstone formed in the Triassic to Cretaceous periods (15), classified into the Khorat geological group (32). The soil is mainly derived from the sandstone of the Phra Wihan formation (31). The area is located on tilted sedimentary layers; therefore, geological conditions differ by area within the region. Red-yellow podzolic soil covers the site and is more than 60 cm deep. These soils are equivalent to Orthic Acrisols, one of the infertile soils of the tropics (15) or Tropustults (62).

The area of the conservation forest (79.6 km^2^) consists mainly of DEF (42.3 km^2^) and DDF (11.8 km^2^), in which tree species in the ectomycorrhizal plant family Dipterocarpaceae were dominant. DEF is a 30–40-m-high dense forest with three vertical strata (66). Saplings of some dipterocarp trees are found on the forest floor, while herbaceous species are rare (34). The total basal area of DEF in the area is 30.2 m^2^ ha^−1^ and the dominant species is *Hopea ferrea* (Dipterocarpaceae) (20). The aboveground biomass of DEF is 394 Mg ha^−1^ in the station (51). There have been no records of forest fires in DEF. DDF is a relatively sparse forest of 20–35-m-high trees. It has one or two vertical strata, and its forest floor is densely covered with the poaceous grass species, *Vietnamosasa pusilla* (56). The total basal area of DDF in the area is 16.5 m^2^ ha^−1^. The forest is dominated by plant species in Dipterocarpaceae, such as *Shorea obtusa*, *S. siamensis*, and *Dipeterocarpus intricatus* (40), which are utilized as sources of timber, fuel wood, and other forest products in Northeast Thailand (40). The aboveground biomass of DDF is 184 Mg ha^−1^ (60). In contrast to DEF, anthropogenic fires from the surrounding farmlands cause annual forest fires in the dry season. A fire-protected dry deciduous forest (FPDDF plot) is located adjacent to DEF and DDF. FPDDF is a small area (Fig. 1A), around which an understory cover of dead plants has been removed, and prescribed burning at the beginning of the dry season has been performed to protect this plot from forest fires since 1967.

### Soil sampling

Soil sampling was conducted in seven existing plots used for the tree census (three DEF (21), three DDF (40), and one FPDDF (22) plots): Although it was considered to be more appropriate to compare FPDDF with DDF in order to evaluate the direct impact of forest fire management, the FPDDF area in Sakaerat was too small to set replicate sampling plots for comparison with DDF plots (Fig. 1). DDF plots were at least 2 km apart from DEF plots and 300 m apart from the FPDDF plot. Each plot of DEF and DDF was at least 150 m and 300 m apart, respectively. Soil sampling was performed in the rainy season in September 2013. Each plot (50×50 m) was comprised of 25 subplots divided in a 5×5 grid with a 10-m interval (Fig. 1B). We collected 10 g of soil twice at less than 10 cm deep and 10 cm in diameter in each 10×10 m subplot with a spade, which was covered with a plastic bag to minimize contamination by fungi among samples. A total of 350 soil samples representing 175 subplots (two replicate samples per sub-plot) were collected across the seven plots. All soil samples were sieved with a 2-mm sieve and then separately placed in a zip-lock plastic bag with silica gel to dry soil and keep minimum humidity during transportation to the laboratory. Soil samples were stored in a freezer at −30°C until processing. All necessary permits for soil transit from Thailand to Japan were issued by the Kobe Plant Protection Station, The Ministry of Agriculture, Forestry and Fisheries of Japan.

### DNA extraction, PCR, and pyrosequencing

After eliminating debris and fine roots, each soil sample (2.0 mL) was inserted into a 15-mL plastic tube with 6.0 mL of cetyl trimethyl ammonium bromide (CTAB) buffer (100 mM Tris-HCl; 1.4 M NaCl; 20 mM EDTA; 2% [w/v] CTAB) and 0.5% (v/v) mercaptoethanol, and total DNA was then extracted as described elsewhere (42). DNA solutions with TE buffer were stored in a freezer at −25°C until PCR.

We analyzed fungal internal transcribed spacer (ITS) sequences for each soil sample based on a tag-encoded massively parallel pyrosequencing procedure (59). PCR was performed to amplify the entire ITS region and the partial ribosomal large subunit region using the fungus-specific high-coverage primer, ITS1F_KYO2 (5′-TAG AGG AAG TAA AAG TCG TAA-3′) (57), and the universal primer, LR3 (5′-CCG TGT TTC AAG ACG GG-3′) (http://www.biology.duke.edu/fungi/mycolab/primers.htm). The PCR cocktail (10 μL in total) consisted of 1 μL of the DNA extract, 0.5 μL of the forward primer and 0.5 μL of the reverse primers (10 μM), 5 μL of 2×Ampdirect Plus buffer (Shimazu, Kyoto, Japan), 0.05 μL BIOTAQ HS DNA Polymerase (Bioline, London, UK), and 2.95 μL of distilled water. We performed PCR using the following thermocycling conditions; at 95°C for the initial 10 min, followed by 25 cycles at 94°C for 30 s, 50°C for 1 min, 72°C for 2 min 30 s, and a final extension at 72°C for 7 min. The PCR product from each soil sample was subjected to a second PCR step that targeted the ITS2 region. Second PCR was conducted using the universal primer ITS3_KYO2 (5′-GAT GAA GAA CGY AGY RAA-3′) (57) fused with 454 Adaptor A and 8-mer sample-specific molecular ID (17); the reverse universal primer was LR_KYO1b (5′-MGC WGC ATT CCC AAA CWA-3′) (58) fused with 454 Adaptor B. A buffer system of Taq DNA Polymerase together with Standard Taq Buffer (New England Biolabs, Ipswich, MA, USA) was used with a temperature profile of 95°C for 10 min, followed by 40 cycles at 94°C for 30 s, 50°C for 1 min, 72°C for 1 min, and a final extension at 72°C for 7 min.

All ITS amplicons of the second PCR steps were pooled and subjected to a purification process by the QIAquick PCR Purification Kit (Qiagen, Netherlands). As instructed by the manufacturer, 454-pyrosequencing was performed using a GS Junior sequencer (Roche, Basel, Switzerland).

### Bioinformatics

In the analysis of the fungal ITS2 region, reads were assembled by Assams v0.1.2012.03.14 (https://www.fifthdimension.jp/products/assams/), which is a highly parallelized extension of the Minimus assembly pipeline (50). Reads were subjected to the *in silico* detection and removal of chimeras (14) (DDBJ DRA accession: DRA006193). Reads in each sample were assembled by Assams with a minimum cut-off similarity of 97% to remove pyrosequencing errors, and chimera reads were then eliminated using the program UCHIME v4.2.40 (14) with a minimum score to report a chimera of 0.1. Within-sample consensus sequences were assembled at a cut-off similarity of 97%, and the output among-sample consensus sequences were used as fungal operational taxonomic units (OTUs; Data S1) in the subsequent procedure. Since OTU sequences reconstructed from a small number of sequencing reads may be derived from sequencing errors, fungal OTUs representing fewer than five reads in at least one sample were excluded in the subsequent analysis. The taxonomic assignment of the remaining OTUs was then conducted based on the query-centric auto-*k*-nearest neighbor method as implemented in Claident v0.1.2012.05.21 (53). The OTUs unassigned to the kingdom Fungi and samples with fewer than 50 reads were eliminated, leaving 830 OTUs from 289 samples (32,492 reads). In order to classify fungal OTUs into functional groups, we used FUNGuild v1.0 (36). On average, 112.4 (SD=38.4; N=289) sequencing reads were obtained from each soil sample.

We then obtained a presence/absence community matrix of fungal OTUs for each soil sample. The number of sequencing reads varied among samples (50–236 reads), which may have artificially generated variance in estimates of *α*-diversity among samples. In order to reduce this bias in downstream analyses, we excluded rare OTUs represented by less than 5% of the total sequencing reads and rarefied the total number of reads of each sample at 50 using the “rrarefy” command of the “vegan” v2.3-0 package (http://www.worldagroforestry.org/publication/vegan-community-ecology-packager-package-vegan-vers-22-1) of R (http://cran.r-project.org/). If fungal OTU data were successfully obtained for both of the replicate samples in each 10×10 m subplot, we used only one of the replicate samples (that with an odd number label). A total of 807 OTUs were detected from 161 subplots.

### Fungal and plant diversity

In order to compare species richness among the forest plots, we analyzed how the number of fungal OTUs increased with higher numbers of samples representing 10×10 m subplots within each 50×50 m forest plot. Since fungal sequencing reads were obtained from less than 20 samples in a DEF plot (DEF1), all samples representing the plot were removed from subsequent analyses. In each plot, we also examined how the number of plant species increased with higher numbers of subplots in order to infer the potential influence of local plant diversity on local fungal diversity. In the diversity analysis of plant communities, we used tree census data of the 10×10 m areas corresponding to the subplots of soil sampling for the fungal diversity analysis in the Sakaerat Environmental Research Station (DEF, DDF, and FPDDF in the latest monitoring period [2006, 2014, and 2014, respectively]) (42). The data of tree individuals with >5 cm in DBH were used in the analysis.

### Fungal community structure

The compositions of fungal OTUs in the soil samples were ordinated using nonmetric multidimensional scaling (NMDS) (23) with two types of dissimilarity indexes, of which one (Raup-Crick dissimilarity [8]) controlled the *α*-diversity of samples and the other (Jaccard dissimilarity) did not. The Raup-Crick and Jaccard indexes were calculated with the “raupcrick” and “vegdist” commands of the vegan package.

We used the presence/absence data of fungal OTUs in each 10×10 m plot. We also investigated whether the OTU compositions of soil fungi varied among the three forest types based on a permutational multivariate analysis of variance (PerMANOVA) (2) using the Raup-Crick/Jaccard dissimilarity index. Data of the sample collected from plot F38 (“Sep_145” in FPDDF) was excluded from this analysis because this sample shared no fungal OTUs with other samples: including the outlier sample may have obscured community compositional differences among other samples. The PerMANOVA analysis was also performed to examine the differentiation of the fungal community structure among replicate plots within the DEF or DDF forest type.

### Relationship between fungal and plant community structures

In order to examine the relationship between plant and soil fungal community structures in each plot, a partial Mantel’s test was performed based on the Raup-Crick/Jaccard dissimilarity of plant and fungal communities, taking into account distances between 10×10 m subplots (10,000 permutations).

We also examined how understory vegetation affected soil fungal community structures. Since the bamboo grass species, *V. pusilla* (Poaceae), dominated the understory vegetation across a broad area of the study forests, we focused on the relationship between the coverage of the plant species and fungal community structures. The coverage of *V. pusilla* was evaluated based on our census with the Braun-blanquet method (33) in each 10×10 m subplot in November 2014. We then examined the relationship between *Vietnamosasa* coverage and the NMDS 1 score in the abovementioned NMDS analysis.

### Habitat preference of fungi

In order to evaluate whether each fungal OTU occurred preferentially in DEF or DDF, we performed the multinomial species classification method (CLAM) (9) using the vegan package. Due to the absence of a replicate plot in FPDDF, we herein focused on DEF and DDF in order to examine the impact of forest fires on fungal community compositions. A CLAM test was performed to classify fungal OTUs into the following four categories: OTUs that preferentially occurred in DEF or DDF, those that frequently occurred in both forest types, and rare fungal OTUs with habitat preferences that were not possible to statistically examine.

All data matrices used in the above statistical analyses are provided in Data S3.

## Results

### Fungal and plant diversity

**Fig. 2:**
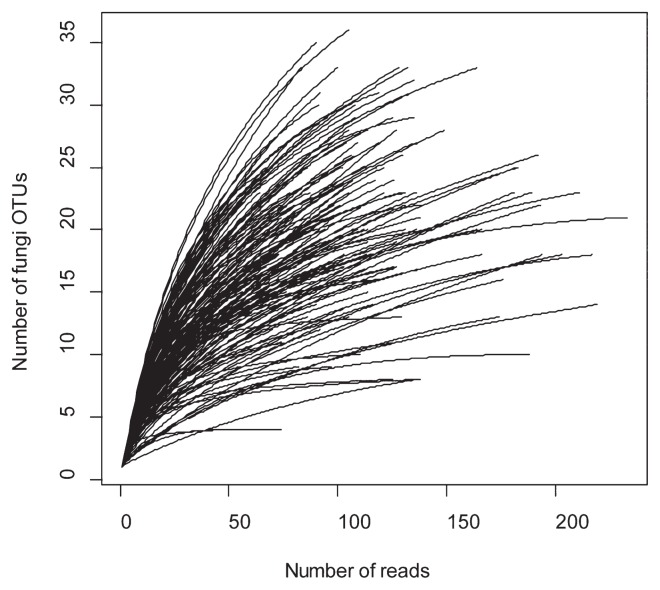
Rarefaction curves of fungal OTUs. In each soil sample, the relationship between the number of pyrosequencing reads, excluding singletons, and the number of fungal OTUs is shown.

**Fig. 3:**
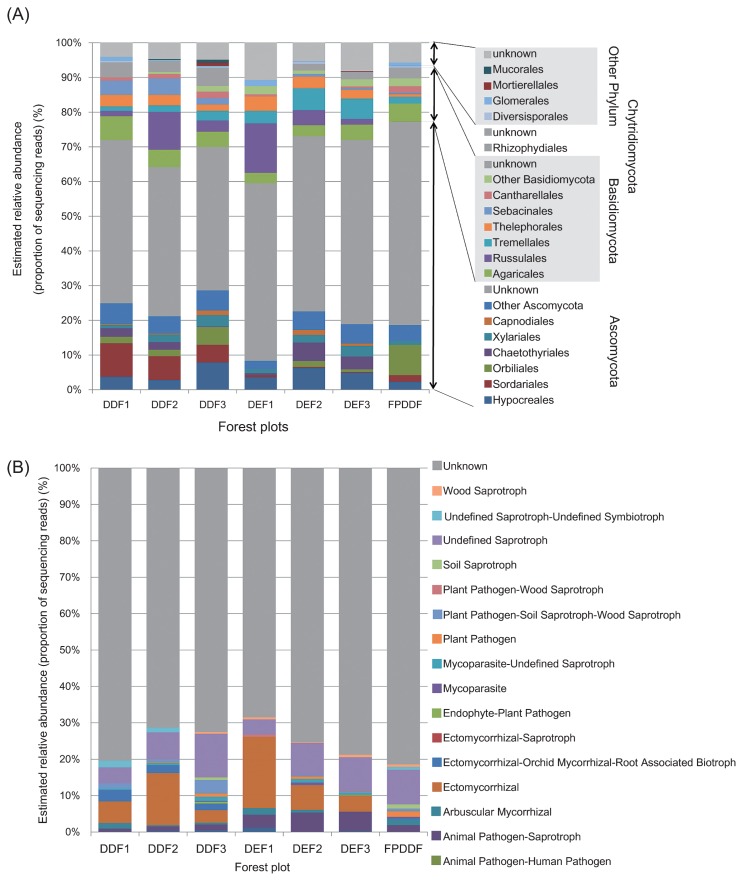
Phylogenetic and functional variations in fungal community compositions across forest plots. (A) Order-level composition of fungal OTUs observed in soil samples collected from seven forest plots. (B) Composition of the fungal functional group (guild) inferred by FUNGuild (36).

**Fig. 4:**
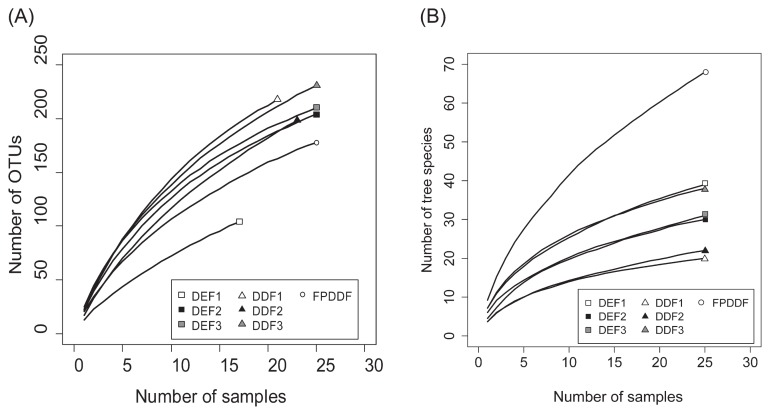
Species accumulation curves. (A) Relationship between the number of subplots and accumulated fungal OTUs within each forest plot. (B) Relationship between the number of subplots and that of tree species. Square: DEF; triangle: DDF; and circle: FPDDF.

**Fig. 5:**
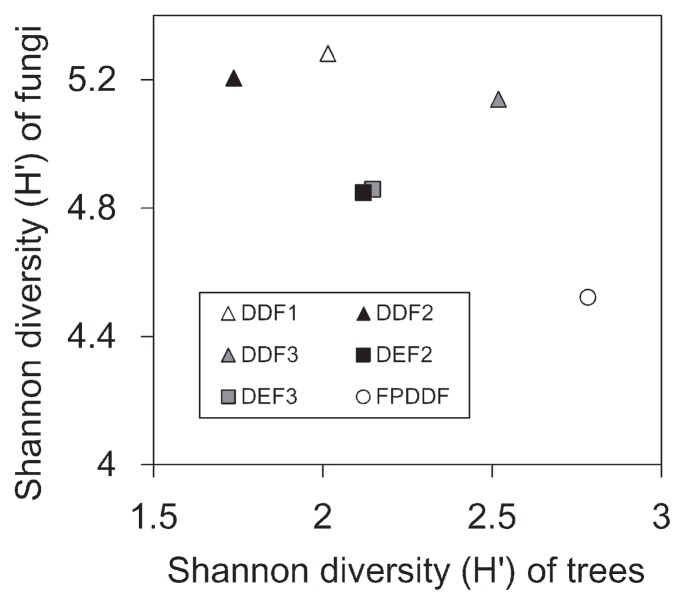
Relationship between plant and fungal diversity. Shannon diversity indices of tree species and fungal OTUs are shown.

The mean number of OTUs observed in each sample was 14.9 (SD=4.9, *N*=288; Fig. 2). Of the 818 fungal OTUs observed across the seven forest plots, 765 (94%) were identified to phylum, 383 (47%) to order, and 201 (25%) to genus (Fig. 3A). At the phylum level, 511 (71.4%) belonged to Ascomycota and 234 (21.5%) were Basidiomycota, followed by 16 (9%) Glomeromycota, and 4 (2%) Chytridiomycota. Although functional groups (guilds) for approximately 70% of the fungal OTUs found in all forest types were unidentified, the compositions of the fungal functional groups varied among the three forest types (Fig. 3B). The total number of observed OTUs increased with higher sample sizes and the rarefaction curve was not saturated, suggesting the high diversity of belowground fungi in the sampling plots (Fig. 4A). Although the species richness of soil fungi did not significantly vary among the forest plots (Wilcoxon rank-sum test with Simpson index; *P*=0.2), the rarefaction curves of DDF plots were above those of the DEF and FPDDF plots (Fig. 4A). On the other hand, the number of tree species was higher in FPDDF than in DEF and DDF (Fig. 4B). The Shannon diversity indexes of the plant and soil fungal communities negatively, rather than positively correlated with each other (*P*=0.0022, Fig. 5).

### Fungal community structure

**Fig. 6:**
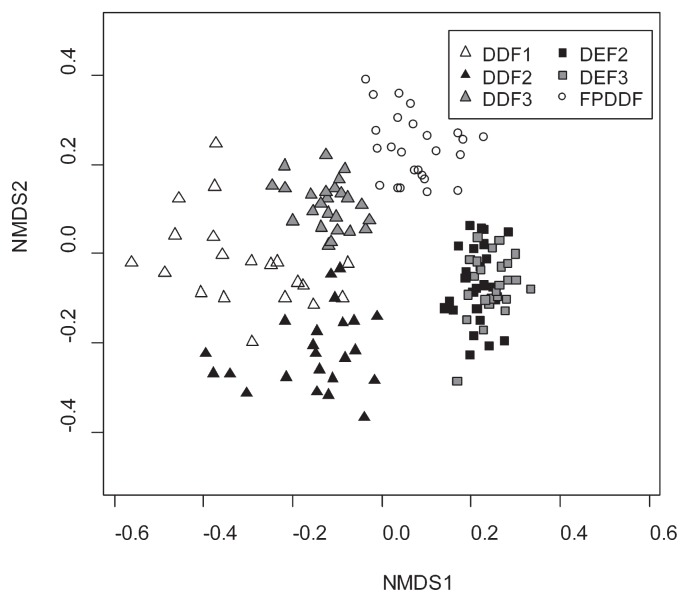
Community structure of soil fungi across forest plots. Nonmetric multidimensional scaling was performed to compare soil fungal community structures among DDF, DEF, and FPDDF plots (stress value=0.1955239). The Raup-Crick dissimilarity index was used. The community structure based on the Jaccard dissimilarity index was shown in Fig. S2.

The community structure of soil fungi was differentiated among the three forest types examined (PerMANOVA, Jaccard: *F*_(2, 141)_=7.29, *P*<0.00001; Raup-Crick: *F*_(2, 141)_=67.58, *P*<0.00001; Fig. 6). We also found that fungal community structures significantly varied among the three DEF plots (Jaccard: *F*_(1, 48)_=1.72, *P*=0.0004; Raup-Crick: *F*_(1, 48)_=6.93, *P*=0.0009) as well as among the three DDF plots (Jaccard: *F*_(2, 66)_=2.58, *P*<0.00001; Raup-Crick: *F*_(2, 66)_=19.45, *P*<0.00001). The fungal OTU compositions in FPDDF were apart from those of DEF and DDF on the NMDS surface (Fig. 6), although 18 OTUs (38.3% of all species in FPDDF) were shared between FPDDF and DDF.

### Relationship between fungal and plant community structures

**Fig. 7:**
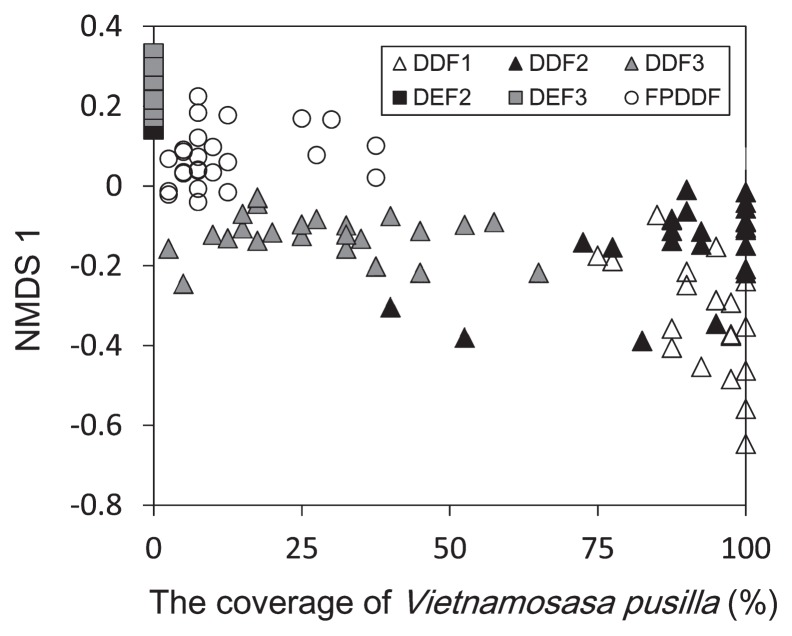
Relationship between *Vietnamosasa* coverage and soil fungal community structure. The coverage of *Vietnamosasa pusilla* and the NMDS 1 score of fungal community structures (Fig. 6) are shown for each subplot.

Tree and fungal community dissimilarity indexes (Raup-Crick/Jaccard dissimilarity estimates) between 10×10 m subplots did not correlate with each other within each plot, even after controlling for spatial distances between subplots (Table 1). In terms of the potential effects of understory vegetation, the coverage of *V. pusilla* strongly correlated with the NMDS 1 axis representing fungal community structures (*r*=−0.79, *P*<0.001; Fig. 7) across the six plots. Even after removing DEF plots, in which *V. pusilla* was absent, a correlation between NMDS 1 scores and *Vietnamosasa* coverage was observed across DDF and FPDDF plots (*r*=−0.63, *P*<0.001).

### Habitat preference of fungi

**Fig. 8:**
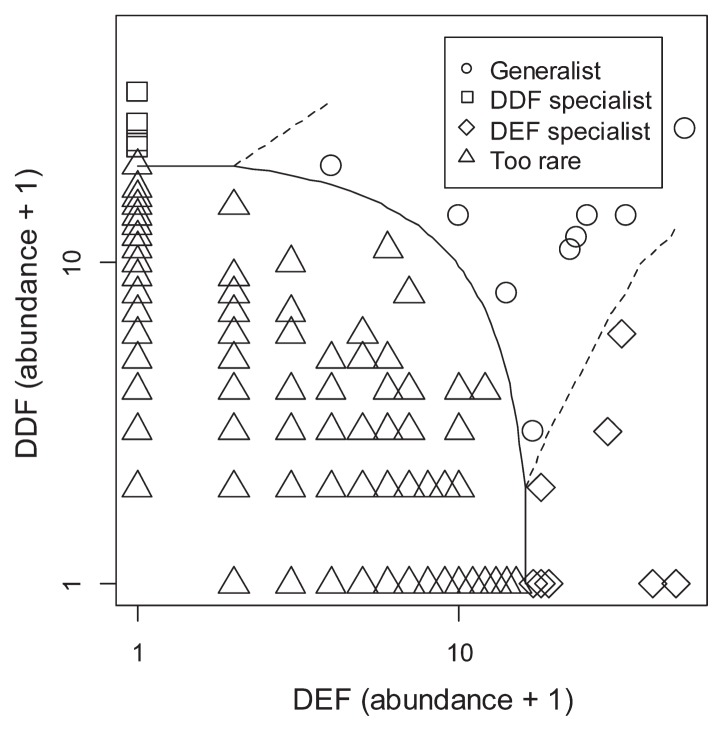
Habitat association of observed fungi. Each fungal OTU is plotted along the axes indicating the time of appearance in soil samples collected from 127 DDF subplots and that in 119 soil samples collected from DEF subplots. Based on the CLAM test (9), each OTU was classified into four categories: fungi showing significant habitat association to DDF plots or DEF plots, those commonly observed in both habitats, and those too rare to evaluate their habitat association.

Of the 741 OTUs found in the DEF and DDF plots, nine OTUs were frequently found in both DEF and DDF (Fig. 8 and Table 2). These fungi with broad habitat ranges involved two Sordaliomycetes (OTU_72 and OTU_627) and unidentified Ascomycota OTUs. The results obtained also showed that 13 OTUs occurred preferentially in DEF plots. Fungi showing preferences for DEF included fungi in diverse taxonomic clades: *e.g.*, *Cryptococcus* sp. (OTU_718), *Gliocephalotrichum* sp. (OTU_611), *Russula* sp. (OTU_613), and *Leohumicola* sp. (OTU_94) as well as fungi in the families Hypocreaceae (OTU_2102) and Herpotrichiellaceae (OTU_440). In contrast, seven OTUs in several Ascomycota clades were found preferentially in DDF plots: *e.g.*, *Fusarium* sp. (OTU_85), Lasiosphaeriaceae (OTU_96), and Orbiliaceae (OTU_432) (Table 2).

## Discussion

Despite the expectation that soil fungal diversity responds to aboveground plant diversity, there was no positive correlation between tree (DBH>5 cm) and fungal diversity indices in the present study; fungal diversity was lower in plots with higher tree diversity (Fig. 5). Previous studies comparing fungal diversity across broad climatic ranges (45, 55) reported that plant species richness in litter does not fully explain soil fungal diversity. Moreover, a study comparing tropical forests showed that high litter plant diversity did not result in high soil fungal diversity (29). Based on these findings, factors other than aboveground plant diversity (28) appear to be responsible for the observed difference in soil fungal diversity among the three forest types. In the Sakaerat Environmental Research Station, the three types of forests differ in the physical and chemical properties of soil (34, 41). For example, seasonally continuous soil erosion is more likely to occur in DDF than in DEF due to forest fires (41). The soil vertical structure to the bedrock was reported to be less developed in DDF than in DEF (34). Moreover, soil aluminum concentrations were markedly higher in DEF than in DDF and FPDDF, while DDF was characterized by higher pH and higher soil calcium and magnesium concentrations (Table S4). In addition, total carbon, total nitrogen, and electric conductivity were higher in FPDDF than in the other two forest types (Table S4). These variations in the physical and chemical characteristics of soil may have contributed to the differentiation of the soil fungal community structure as well as tree community compositions in Sakaerat.

Although tree community diversity did not have the expected effects on soil fungal diversity, the coverage of the shrub bamboo species, *V. pusilla*, strongly correlated with fungal community structures (Fig. 6 and 7). Since the shrub bamboo species was a major source of litter in DDF plots, the understory coverage of the plant may be partly responsible for the differences observed in fungal community structures among the three types of forest plots (DEF, DDF, and FPDDF). After excluding DEF plots, which lacked *V. pusilla* in their understory, the *Vietnamosasa* coverage ratio still strongly correlated with the NMDS 1 axis of the soil fungal community structure. Nevertheless, DDF and FPDDF forests differed in their soil fungal community structures even if they had similar *Vietnamosasa* coverage (Fig. 7), suggesting that fire control since 1967 had a significant impact on fungal communities. Withered *Vietnamosasa* vegetation burned to ashes may provide inorganic nutrients to the ecosystem in DDF, while the removal of understory cover may limit annual dynamic nutrient cycles from aboveground to underground in FPDDF. Thus, contrasting management strategies may result in differences in soil nutrient availability between DDF and FPDDF (Table S4), leading to the differentiation of belowground plant-fungal interactions and fungal functional group compositions.

The community ecological analysis conducted in the present study also allowed us to evaluate the habitat specificity of soil fungi by contrasting the two main components of seasonally dry tropical forests in Sakaerat (*i.e.*, DDF vs. DEF; Fig. 8 and Table 2). Although several fungal OTUs were designated as “habitat generalists” across DEF and DDF, most were unidentified even at the order level (Table 2). This analysis highlighted various fungal taxa showing preferences for DEF, such as an ectomycorrhizal fungus in the genus *Russula* (61), endophytic or saprotrophic fungi in the Ascomycota families in Hypocreaceae and Herpotrichiellaceae (12), and a Nectriaceae fungus in the genus *Gliocephalotrichum*, which causes the fruit rot of rambutan (44). A *Cryptococcus* species showed a significant preference for DEF plots, but was also detected in many DDF and FPDDF plots (Table 2). *Cryptococcus* (OTU_718) was previously reported in various habitats, such as boreal forest soil in the Czech Republic (63), pine forest soil in the Netherlands (47), agricultural field soil in the UK (1), mycorrhizal roots in Germany (38), conifer mycorrhizal roots in Sweden (30), and agricultural soils in Michigan, USA (27). We also found that a fungus in *Leohumicola*, which includes heat-resistant fungi (18), showed a preference for DEF plots, although the biased distribution was not explained by the long-term frequency of forest fires (annual forest fires in DDF vs. no forest fires for >100 years in DEF) in Sakaerat.

While the list of DEF-preferring fungi included both Ascomycota and Basidiomycota, all the fungi showing preferences for DDF plots belonged to Ascomycota (Table 2). Among Ascomycota fungi, *Fusarium* has been reported as plant pathogenic or saprotrophic fungi from forest/agricultural ecosystems worldwide (49). In addition, fungi in the order Sordariales are ubiquitously found from soil and plant debris (6). The analysis also highlighted a fungus in the family Orbiliaceae, which is famous for its nematode-trapping functions (68).

The fungal community composition revealed in the present study was similar to the soil fungal community compositions reported in previous studies in tropical dry forest in Australia, India, Madagascar, and Mexico (55) in some points. At the class level, Agaricomytes, Sordariomycetes, Eurotiomycetes, and Doithideomycetes are common in our and previous studies (57; Fig. S5). On the other hand, the proportion of Tremellomycetes in DEF and that of Orbiliomycetes in FPDDF was higher than the proportions of these fungal taxa in previous studies (55). Meanwhile, the use of 454-sequencing in the present study precluded a thorough understanding of soil fungal diversity, even though we detected hundreds of fungal OTUs. Moreover, although the present study targeted fungal communities, bacterial communities are also expected to play pivotal roles in tropical forest processes and dynamics (13). Thus, for a more comprehensive understanding of variations in soil microbial community structures across seasonally dry tropical and other types of tropical forests, the quality and quantity of raw data need to be enhanced by targeting fungal and bacterial communities and by using more high-throughput sequencing technologies (*e.g.*, Illumina sequencing) in future studies.

Although an increasing number of studies have examined soil fungal diversity (11, 55, 67), limited information is currently available for how forest types are associated with fungal community structures (3). The present study, for the first time, compared fungal community structures between evergreen and deciduous areas in a tropical forest under seasonal risk of forest fires, yielding a list of fungi showing habitat preferences between the forest types. Our results also indicated that even 50-year fire-prevention management resulted in significant compositional changes not only in aboveground tree communities, but also in belowground fungal communities. The development of frameworks for sustainable tropical forest management is an urgent task in the era of global ecosystem degradation. Further studies on soil microbial diversity are required in order to enhance our understanding of how belowground microbial communities and plant-microbe interactions vary depending on forest types and management strategies.

## Supplementary Material



## Figures and Tables

**Table 1:** Test of the relationship between plant and fungal community structures within each forest plot. A partial Mantel’s test was performed based on the Jaccard/Raup-Crick dissimilarities of plant and fungal communities. The spatial distance between 10×10 m subplots was controlled in the analysis.

Forest plot	Jaccard		Raup-Crick	
	
	Mantel’s *r*	*P*	Mantel’s *r*	*P*
DDF1	0.044	0.2851	0.068	0.1867
DDF2	0.0060	0.4712	0.035	0.3348
DDF3	−0.054	0.6961	0.0031	0.4880
FPDDF	−0.049	0.7251	0.031	0.3779
DEF1	−0.13	0.8136	−0.32	0.9964
DEF2	−0.11	0.8588	−0.12	0.8677
DEF3	−0.092	0.7737	−0.17	0.9424

**Table 2:** Habitat specialists and generalists. The fungal OTUs observed preferentially in dry evergreen forest (DEF) or dry deciduous forest (DDF) plots were screened based on a CLAM test. Fungi that occurred commonly in both types of forest plots are shown. The number of DDF, DEF, and FPDDF samples from which each fungal OTU was detected is shown. The trophic mode was inferred using FunGuild (36).

Sample ID	Phylum	Class	Order	Family	Genus	DDF	DEF	FPDDF	Trophic mode
DEF									
OTU_718	Basidiomycota	Tremellomycetes	Tremellales	—	*Cryptococcus*	89	25	6	Pathotroph-Saprotroph
OTU_624	Ascomycota	—	—	—	—	82	0	3	—
OTU_155	Ascomycota	—	—	—	—	72	0	5	—
OTU_607	Ascomycota	—	—	—	—	57	2	0	—
OTU_611	Ascomycota	Sordariomycetes	Hypocreales	Nectriaceae	*Gliocephalotrichum*	38	6	2	Saprotroph
OTU_2101	Ascomycota	Sordariomycetes	Hypocreales	Hypocreaceae	—	23	2	0	Saprotroph
OTU_108	Ascomycota	—	—	—	—	22	0	0	—
OTU_440	Ascomycota	Eurotiomycetes	Chaetothyriales	Herpotrichiellaceae	—	21	0	0	—
OTU_60	—	—	—	—	—	21	0	0	—
OTU_613	Basidiomycota	Agaricomycetes	Russulales	Russulaceae	*Russula*	29	1	1	Symbiotroph
OTU_94	Ascomycota	Leotiomycetes	—	—	*Leohumicola*	20	0	0	Saprotroph
OTU_628	Basidiomycota	Agaricomycetes	Agaricales	—	—	18	0	0	—
OTU_74	Ascomycota	—	—	—	—	18	0	0	—
DDF									
OTU_156	Ascomycota	—	—	—	—	0	42	12	—
OTU_85	Ascomycota	Sordariomycetes	Hypocreales	Nectriaceae	*Fusarium*	0	30	3	Pathotroph-Saprotroph
OTU_626	Ascomycota	—	—	—	—	0	33	0	—
OTU_427	Ascomycota	—	—	—	—	0	29	0	—
OTU_413	Ascomycota	Sordariomycetes	Sordariales	—	—	0	27	0	—
OTU_96	Ascomycota	Sordariomycetes	Sordariales	Lasiosphaeriaceae	—	0	26	0	—
OTU_158	Ascomycota	—	—	—	—	1	24	1	—
OTU_433	Ascomycota	Eurotiomycetes	Onygenales	—	—	0	21	2	—
OTU_432	Ascomycota	Orbiliomycetes	Orbiliales	Orbiliaceae	—	1	18	0	—
Both forest types									
OTU_579	Ascomycota	—	—	—	—	60	16	0	—
OTU_428	Ascomycota	—	—	—	—	35	17	20	—
OTU_430	Ascomycota	—	—	—	—	22	11	14	—
OTU_682	—	—	—	—	—	35	10	0	—
OTU_157	Ascomycota	—	—	—	—	26	13	5	—
OTU_72	Ascomycota	Sordariomycetes	—	—	—	10	14	3	—
OTU_603	Ascomycota	—	—	—	—	3	20	2	—
OTU_627	Ascomycota	Sordariomycetes	Xylariales	—	—	14	7	0	—
OTU_2105	Ascomycota	—	—	—	—	5	14	1	—
