# Supplementary material for: Composition and Diversity of Soil Fungi in Dipterocarpaceae-Dominated Seasonal Tropical Forests in Thailand
Source: Microbes Environ. 2018 May 30;33(2):135–43. doi: 10.1264/jsme2.ME17168 (PMC6031388; doi:10.1264/jsme2.ME17168)
Supplement: Supplementary file 1 [file 33_135_s1.zip › me17168-File013.docx]

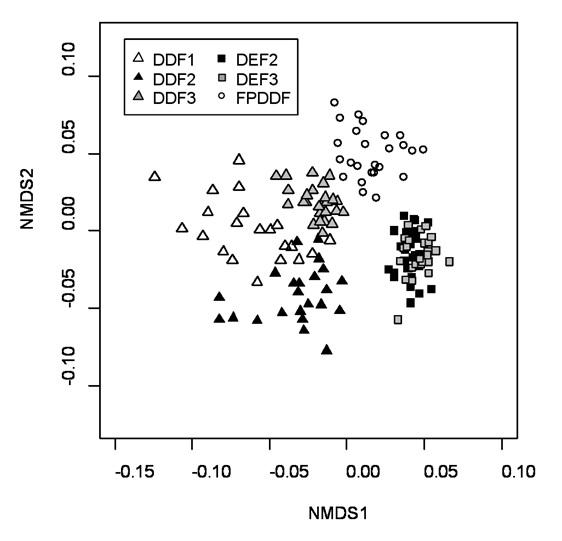


Fig. S2. Community structure of soil fungi across the forest plots (Jaccard dssimilarity).

Sarasa Amma
