# Supplementary material for: Composition and Diversity of Soil Fungi in Dipterocarpaceae-Dominated Seasonal Tropical Forests in Thailand
Source: Microbes Environ. 2018 May 30;33(2):135–43. doi: 10.1264/jsme2.ME17168 (PMC6031388; doi:10.1264/jsme2.ME17168)
Supplement: Supplementary file 1 [file 33_135_s1.zip › me17168-File011.docx]

**Data S1.** Fungal OTU sequences in the FASTA formant.

**Fig. S2.** Community structure of soil fungi across forest plots (Jaccard dissimilarity).

**Data S3.** Data used in each figure.

**Table S4.** Physicochemical properties of soils in three forest types (modified 42).

**Fig. S5.** Class-level composition of fungal OTUs observed in soil samples collected from seven forest plots.
